# Protective Effect of Membrane-Free Stem Cells against Lipopolysaccharide and Interferon-Gamma-Stimulated Inflammatory Responses in RAW 264.7 Macrophages

**DOI:** 10.3390/ijms22136894

**Published:** 2021-06-27

**Authors:** Mei Tong He, Hye Sook Park, Young Sil Kim, Ah Young Lee, Eun Ju Cho

**Affiliations:** 1 Department of Food Science and Nutrition, Pusan National University, Busan 46241, Korea; skyham16@gmail.com (M.T.H.); cando1226@naver.com (H.S.P.); 2 T-Stem Co., Ltd., Changwon 51573, Korea; tstem@t-stem.com; 3 Department of Food Science, Gyeongsang National University, Jinju 52725, Korea

**Keywords:** membrane-free stem cell, anti-inflammatory effect, RAW 264.7 macrophage, lipopolyssacharide, interferon-gamma

## Abstract

Recently, adipose-derived stem cells (ADSCs) are considered to be ideal for application in cell therapy or tissue regeneration, mainly due to their wide availability and easy access. In this study, we examined the anti-inflammatory effects of membrane-free stem cell extract (MFSC-Ex) derived from ADSCs against lipopolysaccharide (LPS)/interferon-gamma (IFN-γ) on RAW 264.7 macrophage cells. Exposure of RAW macrophages to LPS and IFN-γ stimuli induced high levels of nitric oxide (NO), cyclooxygenase-2 (COX-2), and prostaglandin E_2_ (PGE_2_) production. However, pretreatment with MFSC-Ex inhibited LPS/IFN-γ-induced these pro-inflammatory mediators. To clarify the molecular mechanisms underlying the anti-inflammatory property of MFSC-Ex, we analyzed nuclear factor-kappa B (NF-κB) and mitogen-activated protein kinases (MAPKs) protein expressions by Western blotting. Our study showed that treatment of MFSC-Ex significantly down-regulated inducible nitric oxide synthase (iNOS) and COX-2 protein expressions. Furthermore, phosphorylation of extracellular signal-regulated kinase (ERK) and p38 was also blocked by treatment with MFSC-Ex, indicating that inhibitory effect of MFSC-Ex on MAPK signaling cascade may attribute to inactivation of NF-κB. From these findings, we suggest that MFSC-Ex exert anti-inflammatory activities, which suppressed LPS/IFN-γ-induced production of NO, COX-2 and PGE_2_ by regulation of NF-κB and MAPK signaling pathway in RAW 264.7 macrophages. In conclusion, MFSC-Ex might provide a new therapeutic opportunity to treatment of inflammatory-related diseases.

## 1. Introduction

Inflammation is a natural immune reaction in response to harmful stimuli, such as bacteria, viral infection, or toxic compounds [1]. Prolonged inflammatory responses can lead to the development of progressive disorders, including cancer, diabetes, Alzheimer’s disease, and autoimmune conditions. Macrophages play an important role in immune reactions by increasing inflammatory factors and cytokines such as nitric oxide (NO), prostaglandin E_2_ (PGE_2_) interleukin-1beta (IL-1β), IL-6, and tumor necrosis factor-alpha (TNF-α) [2,3]. However, excessive activation of macrophages and pro-inflammatory cytokines are related to pathology of inflammation-related diseases.

Lipopolysaccharide (LPS), which is endotoxin from Gram-negative bacteria, can induce the activation of nuclear factor-kappa B (NF-κB) [4]. Once macrophages are activated by LPS and interferon-gamma (IFN-γ), inhibitor kappa B-alpha (IκB-α) proteins are rapidly phosphorylated and degraded by IκB kinase in the cytoplasm, leading to activation of NF-κB and transcription of corresponding pro-inflammatory mediators, such as inducible nitric oxide synthase (iNOS) and cyclooxygenase-2 (COX-2) [5]. Besides NF-κB activation, the mitogen-activated protein kinases (MAPKs) signaling pathway also takes part in the induction of these pro-inflammatory mediators and cytokines. Macrophages stimulated with LPS lead to MAPK pathways reaching to activation of N-terminal kinase (JNK), extracellular signal-regulated kinase (ERK), and p38 [6,7,8]. It has been shown that suppression of JNK, ERK, and p38 phosphorylation is involved in the decrease of LPS-mediated pro-inflammatory cytokine release [9]. In addition, reduction of these MAPKs expression attenuated NF-κB activation, resulting in protection from LPS-induced injury in tissues [10,11]. Therefore, molecular targeting of NF-κB and MAPK signaling pathways is an effective strategy for the treatment of inflammation-related diseases.

Recently, adipose tissue-derived stem cells (ADSCs) have been applied for the treatment of various diseases due to their ability to regenerate multiple cell types, such as adipocytes, osteoblasts, or neurons [12]. ADSCs are easily isolated from adult adipose tissue, and are capable of modulating inflammation, apoptosis, and angiogenesis [13]. Growing evidence indicated that the beneficial effect of stem cell-based therapy has been attributed to bioactive factors released from stem cells [14,15]. Membrane-free stem cell extract (MFSC-Ex) are produced by the removal of membranes via patented technology from adipose tissue [16]. According to previous studies, MFSC-Ex exert anti-inflammatory, anti-oxidant, and neuroprotective effects in cellular system [17,18,19]. However, the anti-inflammatory effect and underlying mechanisms of MFSC-Ex against LPS and IFN-γ have not been established. Therefore, we examined the protective effect of MFSC-Ex on LPS plus IFN-γ-stimulated NO, PGE_2_, COX-2 production and clarified the mechanisms involved in NF-κB and MAPK signaling pathway in RAW 264.7 macrophages.

## 2. Results

### 2.1. Effect of MFSC-Ex on Cell Viability in LPS/IFN-γ-Stimulated RAW 264.7 Macrophage Cells

**Figure 1:**
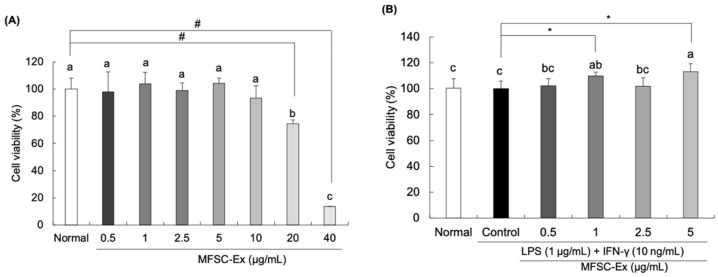
Cytotoxicity of MFSC-Ex on RAW 264.7 macrophage cells in the absence or presence of LPS/IFN-γ. (**A**) Cytotoxic effect of MFSC-Ex on RAW 264.7 macrophage cells. Cells were treated with various concentrations of MFSC-Ex (0.5, 1, 2.5, 5 μg/mL) for 24 h. (**B**) Effect of MFSC-Ex on cell viability LPS/IFN-γ-stimulated RAW 264.7 macrophage cells. Cells were treated with various concentrations of MFSC-Ex (0.5, 1, 2.5, 5 μg/mL) for 2 h, followed by LPS (1 μg/mL) and IFN-γ (10 ng/mL) for 24 h. Values are expressed as means ± SD. ^a–c^ Means with the different letters are significantly different (*p* < 0.05) by Duncan’s multiple range test. ^#^ *p* < 0.05 vs. normal (un-treated) group, * *p* < 0.05 vs. control (LPS/IFN-γ-treated) group.

The effect of MFSC-Ex on LPS/IFN-γ-treated RAW 264.7 macrophage cells was assessed by 3-(4,5-dimethylthiazol-2-yl)-2,5-diphenyl tetrazolium bromide (MTT) assay. Our results showed that MFSC-Ex did not show cytotoxicity at concentrations of 0.5–10 μg/mL (Figure 1A). Therefore, we decided to use the MFSC-Ex extract ranging from 0.5 to 5 μg/mL for subsequent experiments. In addition, there were no statistically significant differences for the LPS/IFN-γ-stimulated and non-stimulated macrophage cell viability (Figure 1B), indicating that LPS and IFN-γ does not have any cytotoxic effects on the cells.

### 2.2. Inhibitory Effect of MFSC-Ex on NO Generation in LPS/IFN-γ-Stimulated RAW 264.7 Macrophage Cells

**Figure 2:**
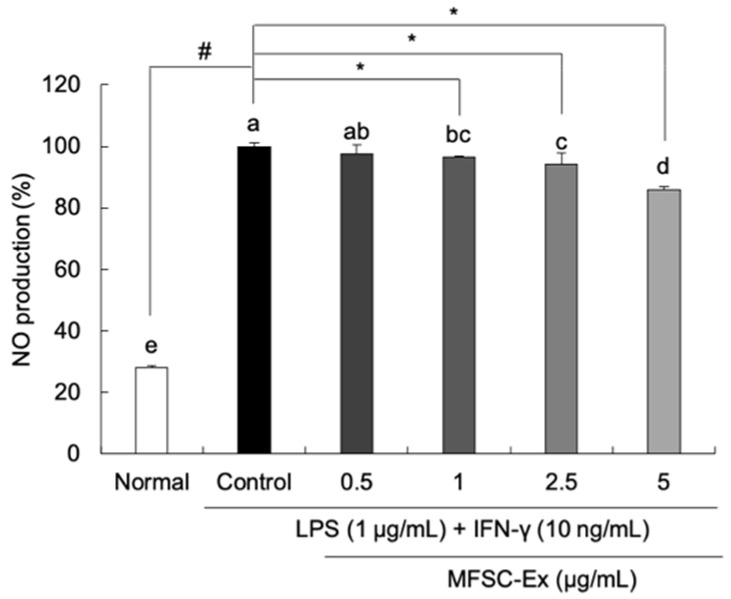
Effect of MFSC-Ex on NO production in LPS/IFN-γ-stimulated RAW 264.7 macrophage cells. Cells were treated with various concentrations of MFSC-Ex (0.5, 1, 2.5, 5 μg/mL) for 2 h, followed by LPS (1 μg/mL) and IFN-γ (10 ng/mL) for 24 h. Values are expressed as means ± SD. ^a–d^ Means with the different letters are significantly different (*p* < 0.05) by Duncan’s multiple range test. ^#^ *p* < 0.05 vs. normal (un-treated) group, * *p* < 0.05 vs. control (LPS/IFN-γ-treated) group.

To examine the protective effect of MFSC-Ex from LPS/IFN-γ-induced NO production, Griess reagent was used. LPS/IFN-γ treatment significantly increased NO production to 100%, as compared to non-treated group (28.08%) (Figure 2). However, MFSC-Ex significantly inhibited the NO production dose-dependently in the LPS/IFN-γ-stimulated macrophages. In particular, MFSC-Ex concentration of 5 μg/mL showed 13.95% inhibition of NO production. Therefore, MFSC-Ex could inhibit LPS/IFN-γ-induced NO production without cytotoxic effect.

### 2.3. Inhibitory Effect of MFSC-Ex on COX-2 and PGE_2_ Generation in LPS/IFN-γ-Stimulated RAW 264.7 Macrophage Cells

**Figure 3:**
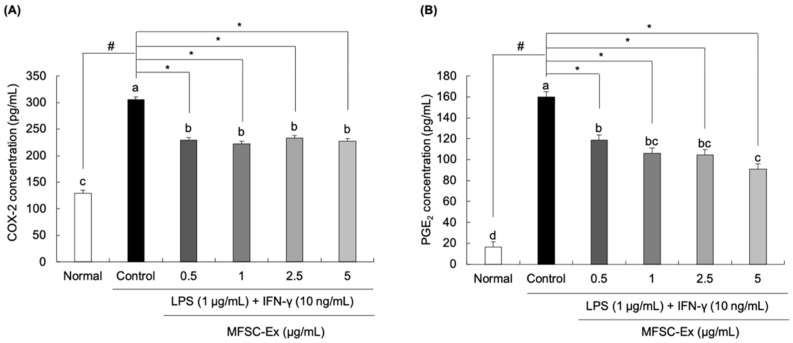
Effect of MFSC-Ex on COX-2 (**A**) and PGE_2_ (**B**) production in LPS/IFN-γ-stimulated RAW 264.7 macrophage cells. Cells were treated with various concentrations of MFSC-Ex (0.5, 1, 2.5, 5 μg/mL) for 2 h, followed by LPS (1 μg/mL) and IFN-γ (10 ng/mL) for 24 h. Values are expressed as means ± SD. ^a–d^ Means with the different letters are significantly different (*p* < 0.05) by Duncan’s multiple range test. ^#^ *p* < 0.05 vs. normal (un-treated) group, * *p* < 0.05 vs. control (LPS/IFN-γ-treated) group.

To investigate the regulatory effects of MFSC-Ex on the production of COX-2 and PGE_2_, we used enzyme-linked immunosorbent assay (ELISA) according to manufacturer’s instruction. In RAW 264.7 macrophage cells exposed to LPS and IFN-γ, the concentration of COX-2 was significantly increased from 129.64 pg/mL to 305.84 pg/mL (Figure 3A). In contrast, the cells treated with MFSC-Ex (at concentrations of 0.5, 1, 2.5, and 5 μg/mL) markedly reduced COX-2 levels by 229.48 pg/mL, 222.76 pg/mL, 232.76 pg/mL, and 227.72 pg/mL, respectively. Further, the PGE_2_ concentrations were also decreased by treatment with MFSC-Ex (Figure 3B), especially MFSC-Ex concentrations of 5 μg/mL effectively suppressed the PGE_2_ production to 90.79 pg/mL, versus only LPS/IFN-γ-stimulated macrophage cells (159.78 pg/mL).

### 2.4. Regulatory Effect of MFSC-Ex on the Protein Levels of iNOS and COX-2 in LPS/IFN-γ-Stimulated RAW 264.7 Macrophage Cell

**Figure 4:**
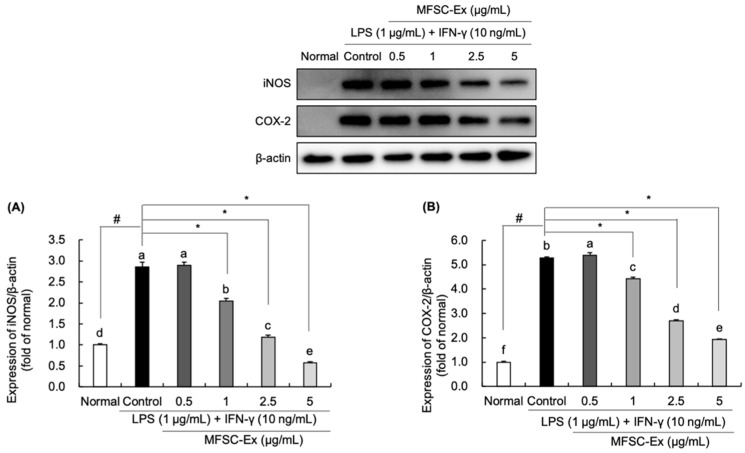
Effect of MFSC-Ex on the levels of iNOS (**A**) and COX-2 (**B**) protein expression in LPS/IFN-γ-stimulated RAW 264.7 macrophage cells. Cells were treated with various concentrations of MFSC-Ex (0.5, 1, 2.5, 5 μg/mL) for 2 h, followed by LPS (1 μg/mL) and IFN-γ (10 ng/mL) for 24 h. Values are expressed as means ± SD. ^a–f^ Means with the different letters are significantly different (*p* < 0.05) by Duncan’s multiple range test. β-actin was used as a internal control for Western blotting. ^#^ *p* < 0.05 vs. normal (un-treated) group, * *p* < 0.05 vs. control (LPS/IFN-γ-treated) group.

To evaluate the potential mechanisms of MFSC-Ex on the inhibition of LPS/IFN-γ-induced NO, COX-2, and PGE_2_ generation, we analyzed iNOS and COX-2 protein expression levels by Western blotting. In our findings, the protein levels of iNOS and COX-2 were considerably increased in LPS/IFN-γ-treated control group, compared to non-treated group (Figure 4A,B). However, MFSC-Ex significantly reduced the expression of iNOS, and it was parallel with the inhibitory effect on NO generation. In addition, a similar tendency of reduction of COX-2 was observed after MFSC-Ex treatment, showing that MFSC-Ex potently suppressed COX-2 protein levels in a concentration-dependent manner. This suppression of iNOS and COX-2 protein levels may be consistent with the inhibitory effect of MFSC-Ex on LPS/IFN-γ-induced NO and PGE_2_ production, respectively.

### 2.5. Regulatory Effect of MFSC-Ex on the Protein Levels of ERK and p-38 Phosphorylation in LPS/IFN-γ-Stimulated RAW 264.7 Macrophage Cell

**Figure 5:**
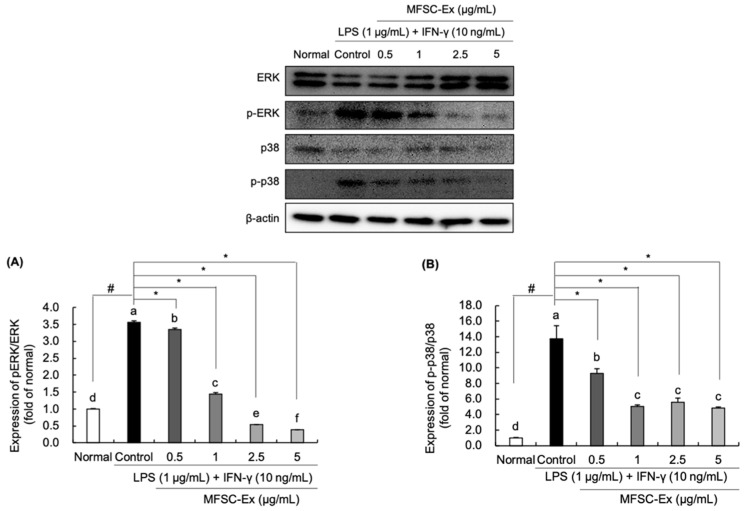
Effect of MFSC-Ex on the levels of phosphorylated-ERK (**A**) and -p38 (**B**) protein expression in LPS/IFN-γ-stimulated RAW 264.7 macrophage cells. Cells were treated with various concentrations of MFSC-Ex (0.5, 1, 2.5, 5 μg/mL) for 2 h, followed by LPS (1 μg/mL) and IFN-γ (10 ng/mL) for 24 h. Values are expressed as means ± SD. ^a–f^ Means with the different letters are significantly different (*p* < 0.05) by Duncan’s multiple range test. β-actin was used as a internal control for Western blotting. *^#^ p* < 0.05 vs. normal (un-treated) group, * *p* < 0.05 vs. control (LPS/IFN-γ-treated) group.

We examined whether these anti-inflammatory properties of MFSC-Ex are associated with MAPK signaling pathway. Our findings exerted that protein expressions of phospho-ERK and -p38 were up-regulated by LPS and IFN-γ stimulation (Figure 5A,B). However, RAW 264.7 macrophages treated with MFSC-Ex significantly decreased phosphorylation levels of ERK and p38. Based on these results, we suggest that MFSC-Ex may disrupt MAPK signaling pathway by ERK and p38 inactivation, resulting in down-regulation of LPS/IFN-γ-mediated iNOS and COX-2 protein expression.

## 3. Discussion

Macrophages play a crucial role in inflammatory responses in the body. When pathogens are detected macrophages provide immune reactions by releasing inflammatory mediators and cytokines [20]. LPS, an endotoxin derived from outer membrane of gram-negative bacterial, is known to be responsible for the inflammatory responses, resulting in the up-regulation of their reactive products, NO and PGE_2_ [21]. In addition, exposure of RAW 264.7 macrophages to LPS with IFN-γ stimuli has been reported to be synergistic that leads to secretion of large amounts of pro-inflammatory cytokines [22]. When macrophage cells were stimulated by LPS and IFN-γ, they modulate the expression of toll-like receptors 4, and subsequently activate NF-κB and MAPK signaling cascade [23]. These two pathways induce the amounts of pro-inflammatory mediators and cytokines release, such as NO, iNOS, COX-2, IL-1β, IL-6, and PGE_2_ [24].

**Figure 6:**
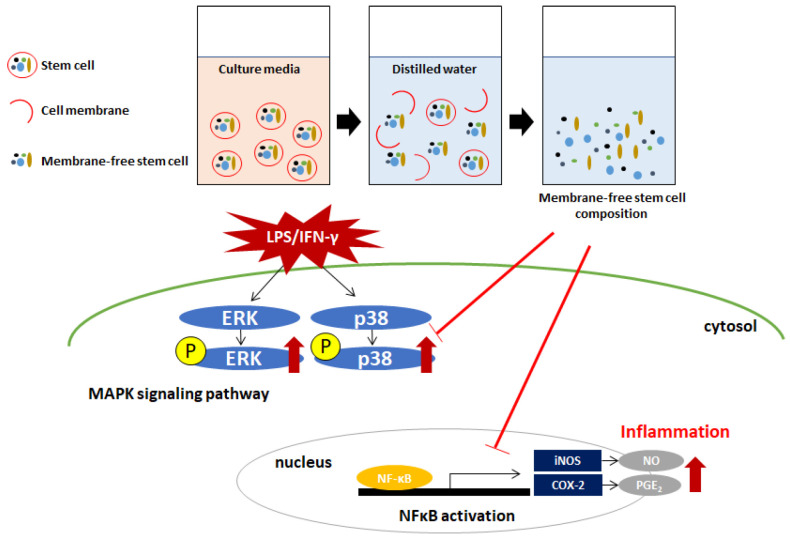
Predicted mechanism on the effect of MFSC-Ex in LPS/IFN-γ-induced RAW 264.7 macrophage cells.

ADSC-based therapy is applicable for treatment of several diseases in these days. It is easily isolated from adipose tissue and has capacity to differentiate into other cell types such as adipocytes, osteoblasts, and neurons [25]. ADSCs have been shown to potent anti-inflammatory activity by decreasing the secretion of pro-inflammatory cytokines, such as IFN-γ and TNF-α [26]. In addition, MFSC-Ex from ADSCs also significantly inhibited the levels of pro-inflammatory mediators and cytokines, such as iNOS, COX-2, NO, and PGE_2_ in rat primary chondrocytes [27]. In this study, we investigated whether MFSC-Ex modulate LPS and IFN-γ-induced inflammatory responses in macrophages (Figure 6).

Firstly, the cytotoxicity of MFSC-Ex on RAW 264.7 macrophage cells was detected by MTT assay. The viability of macrophages treated with 0.5–10 μg/mL was not reduced, suggesting that MFSC-Ex did not exhibit any toxicity in RAW 264.7 cells less than 10 μg/mL. NO is produced from L-arginine, which is oxidized by NOS [28]. NO is one of the most important signaling molecules and plays a critical role in vascular regulation, neural communication, blood clotting, and immune system [29,30]. However, a high concentration of NO in the body leads to oxidative stress and inflammation, resulting in the development of inflammatory-related diseases, such as rheumatoid arthritis, chronic hepatitis, cancer, and neurodegenerative diseases [31,32,33]. Therefore, inhibition of NO is an important target for anti-inflammatory agents. In the current study, we observed that NO generation was significantly increased by LPS/IFN-γ treatment in RAW 264.7 macrophage cells. However, MFSC-Ex significantly inhibited the production of cellular NO dose-dependently, indicating that MFSC-Ex may have anti-inflammatory effect by suppression of NO in macrophage cells.

COX-1 and COX-2 are enzymes that play an important role in regulation of inflammation. COX-1 is expressed ubiquitously and constitutively under normal physiological conditions. In contrast, COX-2 is not constitutively expressed in normal conditions, but it can be induced by endotoxin or pro-inflammatory cytokines, leading to PGE_2_ production [34]. Elevated levels of PGE_2_ were shown to promote inflammatory reactions, thus inhibition of COX-2 can block the PGE_2_ generation [35]. We evaluated the effect of MFSC-Ex on the levels of COX-2 and PGE_2_ concentration in response to LPS/IFN-γ. It was found that COX-2 and PGE_2_ levels were significantly increased by treatment of LPS and IFN-γ, whereas MFSC-Ex significantly suppressed COX-2 and PGE_2_ generation. These results suggest that a decrease of PGE_2_ levels in macrophage cells treated with MFSC-Ex could be due to inhibition of COX-2 production.

To evaluate the potential mechanisms of anti-inflammatory effects against LPS/IFN-γ, the protein expression levels of iNOS and COX-2 were measured by Western blotting. Our result revealed that LPS-induced iNOS and COX-2 protein expressions were down-regulated after MFSC-Ex treatment in a dose-dependent manner. Previous reports also described that ADSCs ameliorate inflammation in renal tissue by decreasing phosphorylated IκB-α and COX-2 expressions [36]. In addition, MFSC-Ex obtained from ADSCs significantly down-regulated protein levels of iNOS and COX-2 in amyloid beta-induced SH-SY5Y neuronal cells [19]. Therefore, it is possible that inhibitory effect of MFSC-Ex on NO, COX-2, and PGE_2_ production may be associated with reduction of iNOS and COX-2 protein expression.

MAPK signaling cascade plays another critical role in cell survival, cell proliferation, apoptosis, and inflammatory responses [37,38]. According to previous studies, ERK and p38 are closely associated with regulation of NF-κB signaling pathway [39,40]. Phosphorylation of ERK and p38 can induce iNOS and COX-2 expressions, leading to NO production [41]. Xagorari et al. [42] reported that luteolin attenuated LPS-induced NO generation via inhibition of both ERK and p38. Our results showed that LPS and IFN-γ stimuli led to phosphorylation of ERK and p38, while pretreatment of MFSC-Ex significantly inhibited LPS/IFN-γ-induced ERK and p38 activation, implying that MFSC-Ex may suppress inflammatory responses by down-regulation of MAPKs. These findings are similar to the previous results that treatment of MFSC-Ex suppressed NF-κB and MAPK signaling pathways in IL-1α-stimulated rat chondrocytes [27]. Therefore, this could explain the mechanism by which MFSC-Ex protect LPS/IFN-γ-mediated iNOS and COX-2 protein expression via regulation of ERK and p38 signaling pathway.

## 4. Materials and Methods

### 4.1. Chemical Reagents

Dulbecco’s modified eagle’s medium (DMEM), fetal bovine serum (FBS), trypsin-ethylenediaminetetraacetic acid (EDTA), and penicillin/streptomycin were obtained from Welgene (Daegu, Korea). The LPS used in this study was from Sigma Chemical Co. (St. Louis, MO, USA), and IFN-γ was purchased from Pepro Tech (Rocky Hill, NJ, USA). The Griess reagent, MTT, and dimethyl sulfoxide (DMSO) were obtained from Sigma Chemical Co. For Western blotting, polyvinylidene fluoride (PVDF) membranes were from Millipore (Billerica, MA, USA), RIPA buffer was acquired from Cell Signaling (Beverly, MA, USA), and pico-enhanced peroxidase detection was obtained from ELPIS-Biotech (Daejeon, Korea).

### 4.2. Preparation of MFSC-Ex

The MFSC-Ex used in this study were produced by T-Stem Co. (Changwon, Korea) [16]. In brief, human fat tissue was obtained from healthy female in her twenties with 2-degree obesity (BMI 25‒29.9), which has been proven to be safe through blood tests. Isolated fat tissues were purified, and the extracted cells were cultured using a serum-free cell culture medium at 37 °C, 5% CO_2_ incubator. Cells were sub-cultured when confluent (70‒80%) until 6–8 passages. After then, stem cells were collected, and the cell membranes were removed by ultrasonication in distilled water. The debris of cells was eliminated from the membranes by centrifugation (at 800–1500 *g*) and then filtered. On the basis of our previous study, the aqueous solution was freeze-dried to obtain powder form and stored at 5 ± 2 °C. The concentration of MFSC-Ex adjusted to various concentrations (0.5, 1, 2.5, 5 μg/mL) using culture medium for cell experiments.

### 4.3. Cell Culture

RAW264.7 macrophage cells were obtained from the Korea Cell Line Bank (KCLB, Seoul, Korea). Cells were grown with DMEM containing 1% penicillin/streptomycin and 10% FBS at 37 °C in a 5% CO_2_ incubator. The cells were sub-cultured weekly with 0.05% trypsin-EDTA in PBS.

### 4.4. Cell Viability

After confluence had been reached, the cells were plated in 96-well plates at a density of 1 × 10^5^ cells/mL and incubated for 24 h. After treatment of LPS (1 μg/mL)/IFN-γ (10 ng/mL) for 2 h, the cells were added with various concentrations of MFSC-Ex (0.5, 1, 2.5, 5 μg/mL) for 24 h. MTT solution was added to each well of the 96-well plate, and the plate was incubated for 4 h at 37 °C, after which the medium containing the MTT was removed. The incorporated formazan crystals in the viable cells were solubilized with DMSO, and the absorbance of each well was read at 540 nm [43].

### 4.5. Measurement of NO Levels

The production of NO was quantified using Griess reagent. Briefly, 100 μL of culture supernatants were collected and mixed with an equal volume of the Griess reagent (0.1% N-(1-naphthyl) ethylenediamine dihydrochloride, 1% sulfanilamide, and 2.5% H_3_PO_4_) at room temperature for 30 min. The absorbance was measured using spectrophotometer at 540 nm. A sodium nitrite (NaNO_2_) standard curve was used to calculate nitrite concentration of cells [44].

### 4.6. Measurement of COX-2 and PGE_2_ Levels

The concentration of COX-2 and PGE_2_ (R&D Systems, Inc., Minneapolis, MN, USA) was measured by ELISA according to manufacturer’s instructions. Briefly, in the measurement of COX-2, samples were added to COX-2 capture antibody-coated plate, and sequentially added detection antibody, streptavidin-HRP A, substrate solution to each well. The absorbance was then read at 450 nm. In the measurement of PGE_2_, cell culture supernatants were added to the plate, and then primary antibody solution, PGE_2_ conjugate, and substrate solution were added to each well in sequence. The absorbance was read at 450 nm.

### 4.7. Western Blotting

The whole cell lysates were prepared according to the manufacturer’s instructions using RIPA buffer supplemented with 1 × protease inhibitor cocktail. The total concentration of protein was determined with bovine serum albumin (BSA) as the standard. Equal amounts of proteins were separated on 10–13% sodium dodecyl sulfate polyacrylamide gel electrophoresis (SDS-PAGE). After electrophoresis, proteins were transferred to PVDF membranes. Membranes were subsequently incubated with 5% skim milk dissolved in phosphate-buffered saline with Tween-20 (PBS-T) for 60 min and further incubated with primary antibody in PBS-T overnight at 4 °C. The primary antibodies were as follows: [iNOS (1:1000, Cell Signaling, Beverly, MA, USA city, state abbrev if USA, country); COX-2 (1:1000, Cell Signaling); ERK 1/2 (1:1000, Cell Signaling); phospho-ERK (1:1000, Cell Signaling); p38 (1:1000, Cell Signaling); phospho-p38 (1:1000, Cell Signaling); and β-actin (1:1000, Cell Signaling)]. After that, the membranes were incubated with appropriate secondary antibodies (1:1000, Cell Signaling) for 1 h. The Western blot bands were visualized by using pico-enhanced peroxidase detection and assessed with a Davinci-Chemiluminescent imaging system (CoreBio, Seoul, Korea).

### 4.8. Statistical Analysis

Results were expressed as means ± SD. Statistical analysis was performed using IBM SPSS statistics software version 23 (IBM Corporation, Armonk, NY, USA). Statistical differences were determined by one-way ANOVA followed by Duncan’s *post-hoc* test (*p* < 0.05).

## 5. Conclusions

Taken together, our findings showed that MFSC-Ex, which is stem cell components without cell membrane, suppressed LPS/IFN-γ-induced NO, COX-2, and PGE_2_ production via down-regulation of iNOS and COX-2 protein expression, and its inhibitory effect might be associated with the decreased protein levels of phospho-ERK and -p38 in RAW 264.7 macrophage cells. Therefore, MFSC-Ex have anti-inflammatory properties in macrophages and may be useful for the prevention or treatment of inflammatory-related disorders as a non-cell-based stem cell therapy.

## Data Availability

The data associated with this research are available and can be obtained by contacting the corresponding author.
